# Evaluation of the impact of assistive artificial intelligence on ultrasound scanning for regional anaesthesia

**DOI:** 10.1016/j.bja.2022.07.049

**Published:** 2022-09-08

**Authors:** James S. Bowness, Alan J.R. Macfarlane, David Burckett-St Laurent, Catherine Harris, Steve Margetts, Megan Morecroft, David Phillips, Tom Rees, Nick Sleep, Asta Vasalauskaite, Simeon West, J. Alison Noble, Helen Higham

**Affiliations:** 1 Oxford Simulation, Teaching and Research Centre, University of Oxford, Oxford, UK; 2 Department of Anaesthesia, Aneurin Bevan University Health Board, Newport, UK; 3 Department of Anaesthesia, Glasgow Royal Infirmary, Glasgow, UK; 4 School of Medicine, Dentistry & Nursing, University of Glasgow, Glasgow, UK; 5 Department of Anaesthesia, Royal Cornwall Hospitals NHS Trust, Truro, UK; 6 Intelligent Ultrasound, Cardiff, UK; 7 Department of Anaesthesia, University College London, London, UK; 8 Institute of Biomedical Engineering, University of Oxford, UK; 9 Department of Anaesthesia, Oxford University Hospitals NHS Foundation Trust, Oxford, UK

**Keywords:** artificial intelligence, peripheral nerve block, regional anaesthesia, sono-anatomy, ultrasonography, ultrasound

## Abstract

**Background:**

Ultrasound-guided regional anaesthesia relies on the visualisation of key landmark, target, and safety structures on ultrasound. However, this can be challenging, particularly for inexperienced practitioners. Artificial intelligence (AI) is increasingly being applied to medical image interpretation, including ultrasound. In this exploratory study, we evaluated ultrasound scanning performance by non-experts in ultrasound-guided regional anaesthesia, with and without the use of an assistive AI device.

**Methods:**

Twenty-one anaesthetists, all non-experts in ultrasound-guided regional anaesthesia, underwent a standardised teaching session in ultrasound scanning for six peripheral nerve blocks. All then performed a scan for each block; half of the scans were performed with AI assistance and half without. Experts assessed acquisition of the correct block view and correct identification of sono-anatomical structures on each view. Participants reported scan confidence, experts provided a global rating score of scan performance, and scans were timed.

**Results:**

Experts assessed 126 ultrasound scans. Participants acquired the correct block view in 56/62 (90.3%) scans with the device compared with 47/62 (75.1%) without (*P*=0.031, two data points lost). Correct identification of sono-anatomical structures on the view was 188/212 (88.8%) with the device compared with 161/208 (77.4%) without (*P*=0.002). There was no significant overall difference in participant confidence, expert global performance score, or scan time.

**Conclusions:**

Use of an assistive AI device was associated with improved ultrasound image acquisition and interpretation. Such technology holds potential to augment performance of ultrasound scanning for regional anaesthesia by non-experts, potentially expanding patient access to these techniques.

**Clinical trial registration:**

NCT05156099.

**Editor's key points**

• Artificial intelligence (AI) applied to ultrasound has the potential to facilitate image acquisition and block placement for regional anaesthesia.
• This exploratory study evaluated ultrasound scanning performance by non-experts with and without use of an AI assistive ultrasound device.
• Use of an AI assistive ultrasound device was associated with improved ultrasound image acquisition and interpretation as judged by experts, and could augment performance by non-experts.

Visualisation of key landmark, target, and safety structures on ultrasonound is a fundamental skill required for the performance of ultrasound-guided regional anaesthesia (UGRA).^1^, ^2^, ^3^ However, the anatomy can be complex and varied^4^, ^5^, ^6^ and, despite ever-improving ultrasound image quality, the ability to acquire or interpret the correct ultrasound view can still be a limiting factor.^7^

Artificial intelligence (AI), particularly in the form of deep learning (DL), is becoming an increasingly mainstream methodology of medical image interpretation. It has been successfully applied to a wide spectrum of medical imaging modalities, including ultrasound.^8^, ^9^, ^10^, ^11^ We and others have reported the use of assistive AI for ultrasound scanning in UGRA.^7^^,^^12^ The device in our studies, ScanNav Anatomy Peripheral Nerve Block (ScanNav^TM^; Intelligent Ultrasound, Cardiff, UK), uses DL techniques to create a real-time colour overlay of B-mode ultrasound to highlight salient structures (Fig 1 and Supplementary File A online video). The system aims to draw attentional gaze to the area of interest, to aid in acquisition of the correct ultrasound view for specific nerve blocks and in the correct identification of structures on that view. We have previously described the potential for this system to achieve high levels of sono-anatomical accuracy with the colour overlay.^13^ Users have reported that it will aid in teaching by experts, and in the learning, performance, and confidence of non-experts in ultrasound scanning for UGRA.^13^ However, there are currently no data comparing scanning performance for UGRA, with and without such a device, to determine whether it directly improves ultrasound scanning.

**Fig 1:**
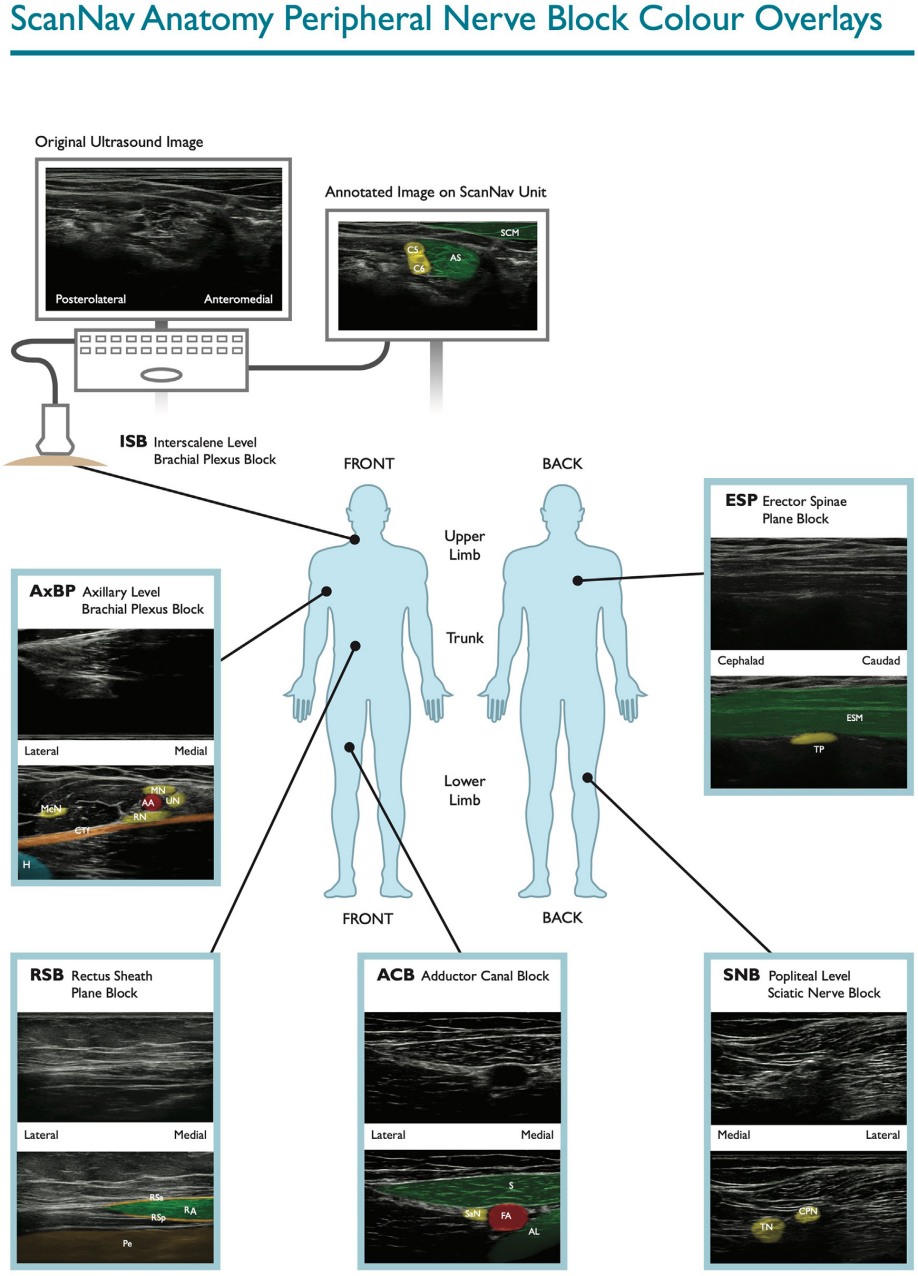
Examples of ScanNav Anatomy Peripheral Nerve Block colour overlay. ISB (interscalene level brachial plexus block): AS, anterior scalene; C5, C5 nerve root; C6, C6 nerve root; MS, middle scalene. AxBP (axillary level brachial plexus block): AA, axillary artery; AV, axillary vein; CT, conjoint (common) tendon of latissimus dorsi/teres major; McN, musculocutaneous nerve; MN, median nerve; RN, radial nerve; UN, ulnar nerve. ESP (erector spinae block): ESM, erector spinae muscle group (and overlying muscles); TP, transverse process. RSB (rectus sheath block): P, peritoneum; RA, rectus abdominis; RSa, rectus sheath (anterior layer); RSp, rectus sheath (posterior layer). ACB (adductor canal block): FA, femoral artery; SaN, saphenous nerve; SM, sartorius muscle. SNB (popliteal level sciatic nerve block): CPN, common peroneal (fibular) nerve; TN, tibial nerve.

Supplementary video related to this article can be found at https://doi.org/10.1016/j.bja.2022.07.049

The following is/are the supplementary data related to this article:

Video 1 Video 1

The present study is a randomised, prospective, interventional study to evaluate the impact of ScanNav^TM^ on the performance of ultrasound scanning for specific blocks by anaesthetists who are non-experts in UGRA. The primary aim of this study was to determine if the use of an assistive AI ultrasound scanning device is associated with improvement in acquisition of the correct block view when performing ultrasound scanning for a peripheral nerve block. The secondary aims were to determine whether use of an assistive AI ultrasound scanning device is associated with improvement in correct identification of sono-anatomical structures on that block view, confidence of the scanner, expert global assessment of scan performance by the non-expert, or time taken for scanning.

## Methods

Ethical approval for this study was granted by the 10.13039/501100000769Oxford University Medical Sciences Inter-Divisional Research Ethics Committee (R70327/RE001). The study was registered with www.clinicaltrials.gov (NCT05156099).

### Non-expert participants

Twenty-one anaesthetists, of varying grades, were recruited from the departments of anaesthesia in Aneurin Bevan University Health Board and Oxford University Hospitals NHS Foundation Trust. All provided written informed consent to participate in this study. All were non-experts in UGRA; none had completed advanced training or a dedicated qualification in UGRA, regularly delivered direct clinical care using UGRA, or regularly delivered teaching on UGRA.

### Expert participants

Six experts in UGRA (AJRM, DBSL, CH, DP, TR, SW) participated in the study, either providing teaching or undertaking assessments of the participants. In accordance with our recent study in this area,^14^ all experts had completed advanced training in UGRA and/or held a dedicated qualification in UGRA, regularly delivered direct clinical care using UGRA (including for ‘awake’ surgery), and regularly delivered teaching on UGRA (including advanced techniques).

### Subjects

Five healthy volunteers for ultrasound scanning were recruited and compensated for their time. The only exclusion criterion was known pathology of the areas to be scanned.

### Equipment

Ultrasound scanning was performed using a linear probe on PX, SII, and X-Porte SonoSite ultrasound machines (Fujifilm SonoSite, Bothell, WA, USA). ScanNav^TM^ was connected to the high-definition multimedia interface output of each ultrasound machine, and stationed next to the machine in question, to display the same ultrasound image but with the associated real-time colour overlay (Fig 1). This device has been given regulatory approval for clinical use in Europe (April 2021) and data collected in this study have been submitted to the notifiable body as part of post-market clinical follow-up activities. The device is not intended to replace clinician judgement and does not make recommendations regarding needle insertion or local anaesthetic injection.

### Teaching

Participants initially attended a 2-h teaching session delivered by three experts (DBSL, CH, TR). A standardised approach to scanning for six of the Plan A Blocks^15^ was taught; interscalene- and axillary-level brachial plexus, erector spinae plane, rectus sheath, adductor canal (distal femoral triangle), and popliteal-level sciatic nerve blocks. This teaching was intended to define a consistent scanning approach amongst participants for each peripheral nerve block, to minimise the influence of varied scanning technique on the outcome measures. The techniques taught were based on identification of the strong recommendation structures for orientation scanning and block view from the recent Regional Anaesthesia UK-led consensus project on ultrasound scanning for basic (Plan A) blocks in UGRA.^16^ One additional structure was included in identification on the block view for the popliteal level sciatic nerve block: the popliteal artery. This teaching session also included familiarisation with ScanNav^TM^ so participants were familiar with the device before assessment.

### Assessment

**Fig 2:**
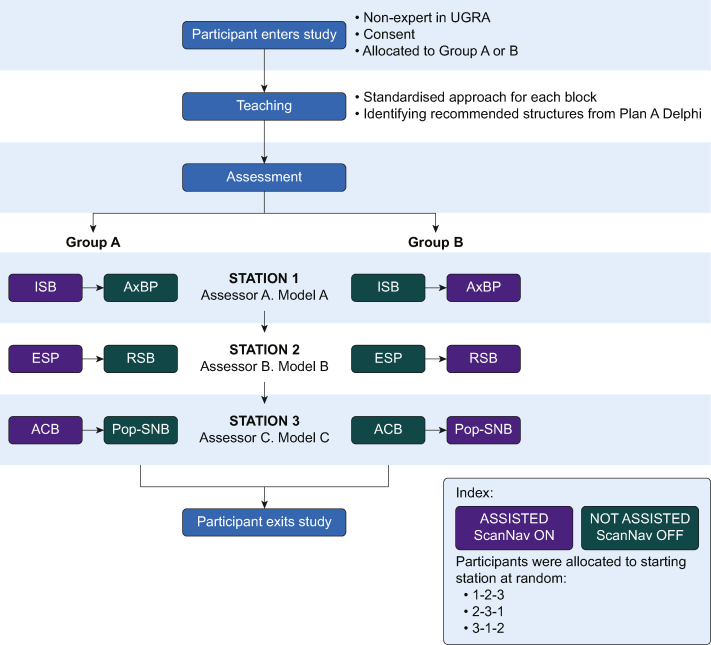
Flow diagram of participant's progress. ACB, adductor canal block; AxBP, axillary level brachial plexus block; ESP, erector spinae plane block; ISB, interscalene level brachial plexus block; Pop-SNB, popliteal level sciatic nerve block; RSB, rectus sheath block; UGRA, ultrasound-guided regional anaesthesia.

Participants performed a single scan for each of the six peripheral nerve blocks whilst being assessed by one of three other experts (AJRM, DP, SW). At the point of recruitment to the study, they were randomised in alternating order based on study enrolment to performing their first scan with or without the device. Participants then alternated between with (aided) and without (unaided) ScanNav^TM^ for six scans, so half of all scans were performed with the aid of the device and half without. Participants were assessed on two of the six scans by an expert assessor performing the scans on a subject. Participants then repeated this process with two other expert/subject pairs (Fig 2). The peripheral nerve block regions for these subjects had not been scanned during the teaching session. The assessors were not blinded to use of the device as it was adjacent to the ultrasound machine during scanning. No expert assessor participated in the earlier teaching or had any previous contribution to the development of ScanNav^TM^.

### Endpoints

Each scan was timed from the point at which ultrasound probe contacted the subject's skin to the point at which the participant declared that they had obtained the correct block view. At this moment the ultrasound image was frozen and the time recorded (seconds). The expert then asked the participant to indicate confidence on a 0–10 scale (0=low confidence, 10=confident). The expert recorded whether the acquired block view was correct (yes/no). A correct block view was defined as one where all the predetermined structures^16^ demonstrated in the earlier teaching sessions were captured on the frozen image. The participant was then asked to identify each strong recommendation structure^16^ for the block view of that particular peripheral nerve block on the original, unmodified image displayed by the SonoSite ultrasound machine. If performing a scan using ScanNav^TM^, participants were allowed to refer to the colour overlay screen to guide their identification on the SonoSite display during the questioning and during the scanning itself. The expert recorded whether structure identification was correct (yes/no). Finally, for that scan, the expert gave the participant a global rating score for that scan on a 0–10 scale (0=poor, 10=excellent). Data points were collected in this order, and participants were not informed of the results of their assessments during this process to avoid influence on performance in individual task or subsequent scans.

### Sample size, data handling, and analysis

The study team was not aware of a similar prior study to inform sample size. Thus, the investigators planned this study to include at least 20 participants as a pragmatic sample that could be adequately managed and provide an indication of the data required to power future studies. Data were recorded manually on data logging sheets, before being transferred to a computer spreadsheet. Data were reported descriptively and, where appropriate, statistical evaluation (using R software V.4.1.1, R Foundation for Statistical Computing, Vienna, Austria^21^) was used to assess the relationship between variables. The χ^2^ test was used to compare discreet data (correct block view, number of structures identified correctly), and the Mann–Whitney *U*-test was used to compare ordinal and non-parametric data (participant confidence, expert global rating score, and scan time). Statistical significance was defined as *P*<0.05. This study was conducted and reported in accordance with the CONSORT-AI guidelines.^17^

## Results

### Participants and subjects scanned

Seven participants were consultant/career grade anaesthetists and 14 were trainees. The three subjects scanned during the assessment phase included a 19-yr-old male (body mass index [BMI] 22.9 kg m^−2^), a 34-yr-old female (BMI 25.1 kg m^−2^), and a 32-yr-old male (BMI 31.7 kg m^−2^).

### Overall data

We performed 126 assessment ultrasound scans (21 for each of the six peripheral nerve block regions); 63 with ScanNav^TM^ and 63 without. During handling, two data points were lost for correct block view (one for a scan with the device and one without) and one data point was lost on scan time (for a scan without the device).

As seen in Table 1, there was an improvement in identification of the correct block view and correct identification of anatomical structures associated with use of ScanNav^TM^. No difference was observed in participant self-rated confidence, expert global rating, or mean scan time.

**Table 1:** Summary of overall endpoints. AI, artificial intelligence; IQR, inter-quartile range; sd, standard deviation.

	Scanning with AI assistive device	Scanning without AI assistive device	Alpha (*P*-value)
Correct block view, *n* (%)	56/62 (90.3)	47/62 (75.1)	0.031
Correct structure identification, *n* (%)	188/212 (88.8)	161/208 (77.4)	0.002
Median confidence (IQR)	8 (6–10)	7 (6–10)	0.155
Median global rating score (IQR)	7 (6–9)	7 (4–9)	0.225
Mean scan time (sd), s	75.9 (69.6)	74.5 (65.6)	0.881

**Fig 3:**
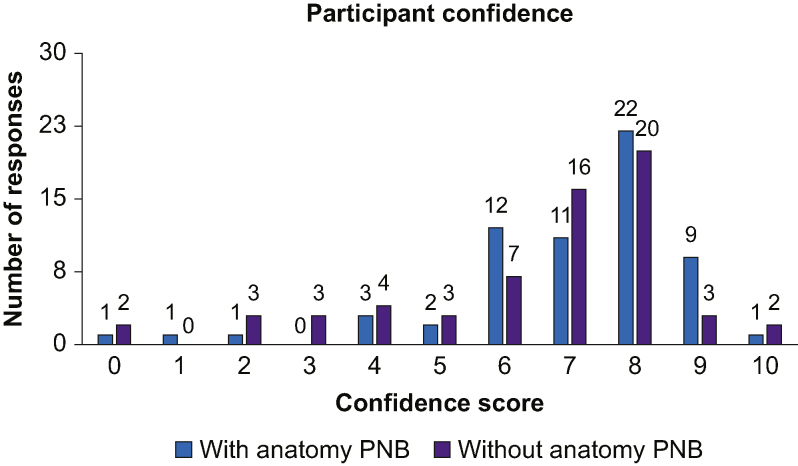
Participant confidence score. Distribution of all participant self-rated confidence scores, showing a breakdown of scans performed with or without ScanNav Anatomy Peripheral Nerve Block. PNB, Peripheral Nerve Block.

**Fig 4:**
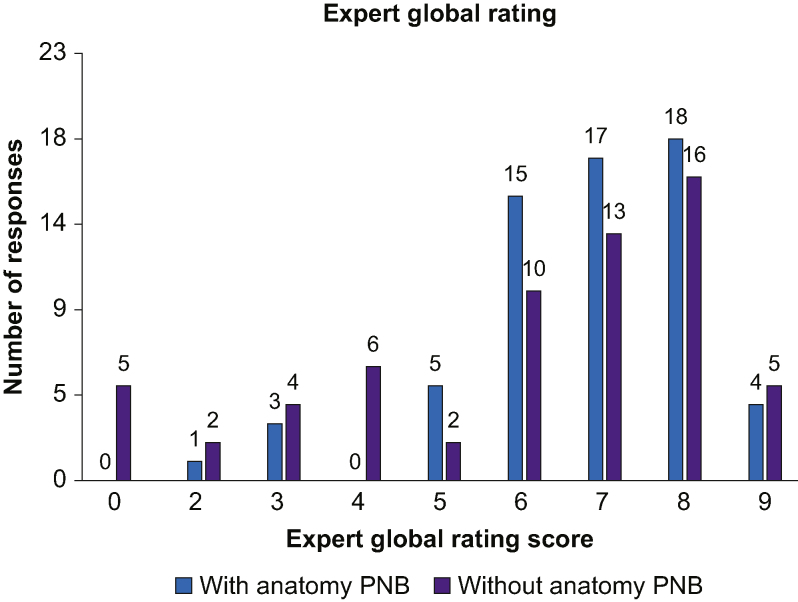
Expert global rating score. Distribution of all expert global rating scores, showing a breakdown of scans performed with or without ScanNav Anatomy Peripheral Nerve Block. PNB, Peripheral Nerve Block.

As seen in Fig 3, participants reported confidence of 0–5 in 23.8% (15/63) of scans without ScanNav^TM^, compared with 12.7% (8/63) when scanning with the device. Conversely, confidence was scored 6–10 in 87.3% (55/63) of scans with compared with 76.2% (48/63) of scans without. Figure 4 shows that experts provided a global rating performance score of 0–5 in 30.2% (19/63) of scans without, compared with 14.3% (9/63) of scans with the device. When considering expert global rating scores of 6–10, 85.7% (54/63) of scans using ScanNav^TM^ achieved this, compared with 69.8% (44/63) without.

### Block-by-block data

Data on individual blocks are not reported here because of small sample size for each peripheral nerve block, but are presented in the Supplementary material. The interscalene level brachial plexus block was the only individual block associated with a significant difference in any endpoint. There was a higher rate of correct anatomical structure identification (*P*<0.001), participant confidence (*P*=0.013), and expert global rating score (*P*=0.013). There was no difference in correct identification of the correct block view or mean scan time for any block individually.

## Discussion

In this exploratory study, the ScanNav^TM^ assistive AI ultrasound scanning device was associated with improved performance by non-experts in UGRA for the acquisition of the correct block view and correct anatomical structure identification. Although the view is not binary (correct/incorrect) in clinical practice, the correct view in this study was based on expert interpretation of ultrasound images containing structures defined by consensus work endorsed by Regional Anaesthesia UK, the American Society of Regional Anesthesia and Pain Medicine, and the European Society of Regional Anaesthesia & Pain Therapy.^16^ The acquisition and interpretation of ultrasound images, despite being an essential skill in UGRA,^1^ is known to be variable, even amongst experts.^18^

This exploratory study shows the potential for assistive AI technology to aid non-experts in one of the fundamental components of UGRA. Such devices could also support the use of UGRA by a wider range of personnel, so enabling improved patient access to the benefits of these techniques.

Improved operator confidence might further facilitate use of UGRA techniques by non-experts and the improved score on expert assessment could be associated with greater readiness for experts to supervise such procedures or allow non-experts greater independence. Although more participants recorded higher scores (≥6/10) in ultrasound scanning confidence and expert global rating with the device than without, the differences do not reach statistical significance. However, both outcomes could facilitate increased patient access to UGRA and further studies should investigate this in detail.

Scanning time was almost identical between the two groups. Our data therefore show that more structures were correctly identified, within the same time, using ScanNav^TM^. The assistive AI technology was not shown to shorten the scanning process, however, mean scan time of around 75 s represents only a small fraction of the perioperative time. Also, a faster scanning process does not mean an improved scanning process. The scanner's workflow therefore does not appear to have been impeded, which might support user acceptance, although this should be specifically addressed in future studies. The AI colour overlay is displayed on a secondary screen; if future generations of ultrasound machine integrate such technology into their primary display, scan times might be reduced if the operator is interacting with only one screen.

Ultrasound scanning is only one aspect of UGRA. However, image acquisition and interpretation inform decision-making for needle insertion and injection. Therefore, optimal performance in these skills is likely to impact efficacy and safety, though this was not investigated in this study. Competence in UGRA is a core learning outcome in the 10.13039/501100001297Royal College of Anaesthetists 2021 training curriculum.^19^ It has been suggested that, to increase utilisation of UGRA techniques by a wider range of anaesthetists, there should be a focus on learning high value, basic (Plan A) blocks rather than more technically challenging or esoteric techniques.^15^ To support this widespread learning and practice, innovation in training and technology is required. These data provide initial evidence that AI technology might be useful for this purpose. Additional studies are needed to assess impact in larger cohorts and on longer term retention of competence, and on determining which clinician group would benefit the most (e.g. novice anaesthetists). The increasing use of point-of-care ultrasound (POCUS) throughout medicine can facilitate implementation of UGRA in appropriate non-anaesthetic specialties (e.g. emergency medicine). Clinicians in these areas, often familiar with POCUS and interventional procedures but less so with UGRA,^20^ could benefit from such technology to support their learning and practice.

The authors recognise imitations to this study. As this is the first work of its kind, the required sample size was unclear in advance. We showed a significant difference in performance between cohorts for the primary endpoint and one of the secondary endpoints. However, based on the above data and assuming a power of 80%, the rate of type 1 (alpha) error in the primary endpoint is calculated at 16%. A larger study would be helpful to confirm or refute these findings and might clarify whether a significant difference exists between other secondary endpoints. Another limitation is that experts were not blinded, which was decided on a pragmatic basis for a number of reasons. Firstly, investigators felt that assessors needed to watch the scanning in real time to assess for atypical anatomy that might influence the presence/absence of structures needed on the block view. An assessor viewing an isolated recording might have greater difficulty placing the images in context and would not be able to see the structures identified on the primary ultrasound screen. Secondly, participants were allowed to refer to the secondary AI screen when identifying structures on the frozen ultrasound image. Thirdly, assessors were asked to give a global rating score of scan performance, which again is more challenging if the assessor views an isolated screen. However, the authors recognise that the objectivity of assessments was limited, which might lead to bias, affecting the internal validity of results and potentially overestimating the effect size of the intervention. Future studies could utilise both a blinded and unblinded assessor, incorporating an assessment of inter-observer variability, to address this. Finally, these data do not include patient outcomes. As the study used healthy volunteers and involved only ultrasound scanning, it is not possible to state that improved acquisition and interpretation of ultrasound images will lead to direct patient benefit, such as improved efficacy or reduced complications.

The authors would like to conclude the discussion by confirming in detail the industrial affiliation in this study. As data from this study will also contribute to regulatory review processes in Europe, employees of the company producing ScanNav^TM^ and funding this study contributed to this work (SM, MM, NS, AV). Other authors have previously or continue to undertake advisory/consulting work (JSB, DBSL, AJRM, JAN, DP). The study concept and design principally fell to clinicians (JSB, DBSL, AJRM) with input from company employees which informed conduct of the study. Data collection was not performed by company employees, whilst analysis and presentation was jointly performed by multiple authors.

### Conclusions

This is the first study that directly compares ultrasound scanning for regional anaesthesia, with and without an assistive AI device, by non-experts. Use of ScanNav Anatomy Peripheral Nerve Block was associated with improved ultrasound image acquisition and interpretation. We conclude that such technology might be used in the future to augment performance by non-experts and potentially expand patient access to these techniques.

## Authors' contributions

Study concept and design: JSB, DBSL, AJRM, SM, MM, NS, AV

Participant recruitment and data collection: JSB, DBSL, CH, HH, AJRM, MM, DP, TR, AV, SW

Data analysis and manuscript preparation: JSB, MM

Manuscript review, editing and approval: all authors

## Declarations of interest

JSB is a Senior Clinical Advisor for Intelligent Ultrasound, receiving research funding and honoraria. DBSL is a Clinical Advisor for Intelligent Ultrasound, receiving honoraria. AJRM is the President of Regional Anaesthesia UK and has received honoraria from Intelligent Ultrasound. DP has received honoraria from Intelligent Ultrasound. SW is board member of Regional Anaesthesia UK. JAN is a Senior Scientific Advisor for Intelligent Ultrasound. Data from this study have been included in European medical device regulatory approval submissions. The device studied (ScanNav Anatomy Peripheral Nerve Block) is a product of Intelligent Ultrasound.

## Funding

Intelligent Ultrasound Limited (Cardiff, UK) via a grant to JSB administered by the University of Oxford (R70327/CN002). JSB has also received a National Institute of Academic Anaesthesia/Barema joint research grant (HMR03690/HM00.01).
