# Supplementary material for: Evaluation of the impact of assistive artificial intelligence on ultrasound scanning for regional anaesthesia
Source: Br J Anaesth. 2022 Sep 8;130(2):226–33. doi: 10.1016/j.bja.2022.07.049 (PMC9900732; doi:10.1016/j.bja.2022.07.049)
Supplement: Multimedia component 2 [file mmc2.docx]

# Supplementary File B

## Healthy volunteer Subjects

(*Regions of body scanned in teaching phase different to assessment phase)

| **Phase** | **Day 1** | **Day 2** |
| --- | --- | --- |
| **Teaching** | M1 M2 M3 | M1 M2 M3 |
| **Assessment** | M4 M5 M3* | M4 M5 M3* |

| **Model ID** | **Sex** | **Age (yrs)** | **BMI (kg/m^2^)** |
| --- | --- | --- | --- |
| M1 | Male | 52 | 22.35 |
| M2 | Male | 69 | 30.06 |
| M3 | Male | 19 | 22.87 |
| M4 | Female | 34 | 25.15 |
| M5 | Male | 32 | 31.70 |

## Assessment - Anatomical structures of interest

| **Item** | **Anatomical Region** | A**natomical structures** |
| --- | --- | --- |
|  | Interscalene level brachial plexus  (Four structures on block view) | - *Scalenus anterior* - *Scalenus medius* - *C5 nerve root* - *C6 nerve root* |
|  | Axillary level brachial plexus  (Six structures on block view) | - *Axillary artery* - *Musculocutaneous nerve* - *Median nerve* - *Ulnar nerve* - *Radial nerve* - *Conjoint tendon* |
|  | Erector Spinae Plane (ESP)  (Two structures on block view) | - *ESP muscle group* - *Transverse processes* |
|  | Rectus sheath  (Three structures on block view) | - *Peritoneum/contents* - *Rectus sheath* - *Rectus abdominis muscle* |
|  | Adductor canal  (Three structures on block view) | - *Femoral artery* - *Saphenous nerve* - *Sartorius muscle* |
|  | Popliteal level sciatic nerve  (Two structures on block view) | - *Sciatic nerve* - *Popliteal artery* |

# Participant background

| **Participant ID** | **Gender** | **Level** | **Length of experience in UGRA** | **Number UGRA procedures performed per year** |
| --- | --- | --- | --- | --- |
| P1 | Male | Trainee | Under 1 year | Under 10 |
| P2 | Female | Con/Career Grade | 3-5 years | Under 10 |
| P3 | Female | Con/Career Grade | 3-5 years | Under 50 |
| P4 | Female | Trainee | 3-5 years | Under 10 |
| P6 | Male | Trainee | Under 1 year | Under 10 |
| P7 | Male | Trainee | 1-3 years | Under 50 |
| P10 | Male | Trainee | Under 1 year | Under 50 |
| P11 | Female | Trainee | 1-3 years | Under 50 |
| P12 | Male | Trainee | Under 1 year | Under 50 |
| P13 | Female | Con/Career Grade | Under 1 year | Under 10 |
| P14 | Female | Con/Career Grade | Over 10 years | Under 50 |
| P15 | Male | Con/Career Grade | 3-5 years | Under 50 |
| P16 | Male | Con/Career Grade | 5-10 years | Under 50 |
| P17 | Male | Con/Career Grade | Under 1 year | Under 10 |
| P18 | Female | Trainee | 1-3 years | Under 10 |
| P19 | Female | Trainee | Under 1 year | Under 10 |
| P20 | Female | Trainee | Under 1 year | Under 10 |
| P21 | Male | Trainee | 1-3 years | Under 50 |
| P22 | Male | Trainee | 1-3 years | Under 50 |
| P23 | Male | Trainee | 3-5 years | Under 50 |
| P24 | Male | Trainee | Under 1 year | Under 10 |

# Study Results

## Identification of the correct block site (expert assessor opinion)

| **Correct Identification of block site (Y/N)** | **With Anatomy PNB** | **Without Anatomy PNB** | | **TOTAL SCANS** |
| --- | --- | --- | --- | --- |
| **Yes** | 56 (90.3%) | 47 (75.1%) | 103 |  |
| **No** | 6 (9.7%) | 15 (24.9%) | 21 |  |
| **TOTAL SCANS** | 62 | 62 | 124 |  |

Participants showed a statistically significant increase in identification of the correct block site when using Anatomy PNB for their scan (Chi-square test, p = 0.0312).

N.B. Two data points lost, thus n = 62 (with and without Anatomy PNB)

### *Identification of the correct block site per block*

| **Block** | **p Value (Chi-square test)** |
| --- | --- |
| **Axillary** | 0.9289 |
| **Interscalene** | 0.2392 |
| **Rectus Sheath** | 0.9610 |
| **ESP** | 0.8273 |
| **Adductor Canal** | 1 |
| **Popliteal** | 0.3484 |

##

## Identification rates of all anatomical structures

|  | **With Anatomy PNB** | **Without Anatomy PNB** | **total scans** |
| --- | --- | --- | --- |
| **Structures identified correctly** | 188 (88.8%) | 161 (77.4%) | 349 |
| **Structures identified incorrectly** | 24 (11.3%) | 47 (22.6%) | 71 |
| **Total scans** | 212 | 208 | 420 |

The use of Anatomy PNB was associated with a statistically significant increased correct identification of key anatomical structures, and lower error rate (Chi-square test, p = 0.0021). Additionally, on a per-block level, use of Anatomy PNB was associated with an increased correct anatomical structure identification rate for the interscalene level brachial plexus block (Chi-square test, p = 0.0001).

### *Identification rates of all anatomical structures by block*

| **Block** | **p Value (Chi-square test)** |
| --- | --- |
| **Axillary** | 0.3540 |
| **Interscalene** | 0.0001 |
| **Rectus Sheath** | N/A* |
| **ESP** | N/A* |
| **Adductor Canal** | 0.9215 |
| **Popliteal** | 0.9215 |

*group performance was identical

## Expert observer’s global scan rating

| **Global Score** | **WITH ANATOMY PNB** | **WITHOUT ANATOMY PNB** | **TOTAL SCANS** |
| --- | --- | --- | --- |
| **0** | 0 | 5 | 5 |
| **2** | 1 | 2 | 3 |
| **3** | 3 | 4 | 7 |
| **4** | 0 | 6 | 6 |
| **5** | 5 | 2 | 7 |
| **6** | 15 | 10 | 25 |
| **7** | 17 | 13 | 30 |
| **8** | 18 | 16 | 34 |
| **9** | 4 | 5 | 9 |
| **tOTAL Scans** | 63 | 63 | 126 |
| **Median** | 7 | 7 |  |
| **IQR** | 6 - 9 | 4 - 9 |  |

Overall, the use of Anatomy PNB was not associated with a significant change in global scan score (Mann-Whitney U test, p = 0.2249).

On a per-block basis, the use of Anatomy PNB was associated with a significant increase in global scan score for the interscalene level brachial plexus block where the median score increased from 4.0 without Anatomy PNB, to 7.0 with Anatomy PNB (Mann-Whitney U test, p = 0.0128).

## Participant confidence

| **Confidence Score** | **WITH ANATOMY PNB** | **WITHOUT ANATOMY PNB** | **TOTAL SCANS** |
| --- | --- | --- | --- |
| **0** | 1 | 2 | 3 |
| **1** | 1 | 0 | 1 |
| **2** | 1 | 3 | 4 |
| **3** | 0 | 3 | 3 |
| **4** | 3 | 4 | 7 |
| **5** | 2 | 3 | 5 |
| **6** | 12 | 7 | 19 |
| **7** | 11 | 16 | 27 |
| **8** | 22 | 20 | 42 |
| **9** | 9 | 3 | 12 |
| **10** | 1 | 2 | 3 |
| **TOTAL SCANS** | 63 | 63 | 126 |
| **Median** | 8 | 7 |  |
| **IQR** | 6 - 10 | 6 - 10 |  |

Whilst overall average and median self-rated confidence scores increased with the use of Anatomy PNB, this was not found to be statistically significant (Mann-Whitney U test, p = 0.1548). However, Anatomy PNB use was associated with a significant increase in participant self-rated confidence whilst scanning for the interscalene level brachial plexus block (Mann-Whitney U test, p = 0.0128).

## Scan Time

| **Anatomy PNB Guided (Y/N)** | **Mean Scan Time**  **(s)** | **Standard Deviation** | **No.** |
| --- | --- | --- | --- |
| **Yes** | 75.92 | ±69.65 | 63 |
| **No** | 74.53 | ±65.62 | 62 |
| **Total** | | | 125 |

Anatomy PNB was not associated with a significant change in the average scan time for participants to identify their chosen block site (Mann-Witney U test, p = 0.88076).

N.B. One data point lost, thus n – 62 for scans without Anatomy PNB.

# Results by block

## Correct identification of block site – by block

### Interscalene

| **Correct Identification of block site (Y/N)** | **With Anatomy PNB** | **Without Anatomy PNB** | **TOTAL SCANS** |
| --- | --- | --- | --- |
| **Yes** | 8 (88.9%) | 6 (54.5%) | 14 |
| **No** | 1 (11.1%) | 5 (45.5%) | 6 |
| **TOTAL SCANS** | 9 | 11 | 20 |

A chi-square test of independence showed that there was no statistically significant association between use of Anatomy PNB and identification of the correct block site (X^2^ = 1.3853, df=1, N = 20, p = 0.2392).

### Axillary

| **Correct Identification of block site (Y/N)** | **With Anatomy PNB** | **Without Anatomy PNB** | **TOTAL SCANS** |
| --- | --- | --- | --- |
| **Yes** | 10 (90.9%) | 8 (80.0%) | 18 |
| **No** | 1 (9.1%) | 2 (20.0%) | 3 |
| **TOTAL SCANS** | 11 | 10 | 21 |

A chi-square test of independence showed that there was no statistically significant association between use of Anatomy PNB and identification of the correct block site (X^2^ = 0.0080, df=1, N = 21, p = 0.9289).

### ESP

| **Correct Identification of block site (Y/N)** | **With Anatomy PNB** | **Without Anatomy PNB** | **TOTAL SCANS** |
| --- | --- | --- | --- |
| **Yes** | 10 (100%) | 11 (100%) | 21 |
| **No** | 0 (0%) | 0 (0%) | 0 |
| **TOTAL SCANS** | 10 | 11 | 21 |

A chi-square test of independence showed that there was no statistically significant association between use of Anatomy PNB and identification of the correct block site (X^2^ = 0.0476, df=1, N = 21, p = 0.8273).

### Rectus Sheath

| **Correct Identification of block site (Y/N)** | **With Anatomy PNB** | **Without Anatomy PNB** | **TOTAL SCANS** |
| --- | --- | --- | --- |
| **Yes** | 11 (100%) | 9 (90.0%) | 20 |
| **No** | 0 (0%) | 1 (10.0%) | 1 |
| **TOTAL SCANS** | 11 | 10 | 21 |

A chi-square test of independence showed that there was no statistically significant association between use of Anatomy PNB and identification of the correct block site (X^2^ = 0.0024, df=1, N = 21, p = 0.9610).

### Adductor Canal

| **Correct Identification of block site (Y/N)** | **With Anatomy PNB** | **Without Anatomy PNB** | **TOTAL SCANS** |
| --- | --- | --- | --- |
| **Yes** | 9 (81.8%) | 8 (80.0%) | 17 |
| **No** | 2 (18.2%) | 2 (20.0%) | 4 |
| **TOTAL SCANS** | 11 | 10 | 21 |

A chi-square test of independence showed that there was no statistically significant association between use of Anatomy PNB and identification of the correct block (X^2^ = 1.9766x10^-31^, df=1, N = 21, p = 1).

### Popliteal

| **Correct Identification of block site (Y/N)** | **With Anatomy PNB** | **Without Anatomy PNB** | **TOTAL SCANS** |
| --- | --- | --- | --- |
| **Yes** | 8 (80.0%) | 5 (50.0%) | 13 |
| **No** | 2 (20.0%) | 5 (50.0%) | 7 |
| **TOTAL SCANS** | 10 | 10 | 20 |

A chi-square test of independence showed that there was no statistically significant association between use of Anatomy PNB and identification of the correct block site (X^2^ = 0.8791, df=1, N = 20, p = 0.3484).

## Identification rates of anatomical structures of interest – by block

### Interscalene

|  | **With Anatomy PNB** | **Without Anatomy PNB** | **TOTAL STRUCTURES** |
| --- | --- | --- | --- |
| **Correctly identified key structures** | 35 (87.5%) | 21 (47.7%) | 56 |
| **incorrectly identified key structures** | 5 (12.5%) | 23 (52.3%) | 28 |
| **TOTAL STRUCTURES** | 40 | 44 | 84 |

A chi-square test of independence showed that there was a statistically significant association between use of Anatomy PNB and correct identification of anatomical landmarks (X^2^ = 14.9148, df=1, N = 84, p = 0.0001).

### Axillary

|  | **With Anatomy PNB** | **Without Anatomy PNB** | **Total structures** |
| --- | --- | --- | --- |
| **Correctly identified key structures** | 52 (78.8%) | 43 (71.7%) | 95 |
| **incorrectly identified key structures** | 14 (21.2%) | 17 (28.3%) | 31 |
| **TOTAL STRUCTURES** | 66 | 60 | 126 |

A chi-square test of independence showed that there was no statistically significant association between use of Anatomy PNB and correct identification of anatomical landmarks (X^2^ = 0.8592, df=1, N = 126, p = 0.3540).

### ESP

|  | **With Anatomy PNB** | **Without Anatomy PNB** | **TOTAL STRUCTURES** |
| --- | --- | --- | --- |
| **Correctly identified key structures** | 20 (100%) | 22 (100%) | 42 |
| **incorrectly identified key structures** | 0 (0%) | 0 (0%) | 0 |
| **TOTAL STRUCTURES** | 20 | 22 | 42 |

Both groups performed equally, with no incorrect identifications.

### Rectus Sheath

|  | **With Anatomy PNB** | **Without Anatomy PNB** | **TOTAL STRUCTURES** |
| --- | --- | --- | --- |
| **Correctly identified key structures** | 33 (100%) | 30 (100%) | 63 |
| **incorrectly identified key structures** | 0 (0%) | 0 (0%) | 0 |
| **TOTAL STRUCTURES** | 33 | 30 | 63 |

Both groups performed equally, with no incorrect identifications.

### Adductor Canal

|  | **With Anatomy PNB** | **Without Anatomy PNB** | **TOTAL STRUCTURES** |
| --- | --- | --- | --- |
| **Correctly identified key structures** | 31 (93.9%) | 28 (93.3%) | 59 |
| **incorrectly identified key structures** | 2 (6.1%) | 2 (6.7%) | 4 |
| **TOTAL STRUCTURES** | 33 | 30 | 63 |

A chi-square test of independence showed that there was no statistically significant association between use of Anatomy PNB and correct identification of anatomical landmarks (X^2^ = 0.0097, df=1, N = 63, p = 0.9215).

### Popliteal

|  | **With Anatomy PNB** | **Without Anatomy PNB** | **TOTAL STRUCTURES** |
| --- | --- | --- | --- |
| **Correctly identified key structures** | 17 (85.0%) | 17 (77.3%) | 20 |
| **incorrectly identified key structures** | 3 (15.0%) | 5 (22.7%) | 22 |
| **TOTAL STRUCTURES** | 20 | 22 | 42 |

A chi-square test of independence showed that there was no statistically significant association between use of Anatomy PNB and correct identification of anatomical landmarks (X^2^ = 0.4057, df=1, N = 43, p = 0.5242).

## Time – by block

| **Block** | **Scan time (s) With Anatomy PNB** | **Scan Time (s)**  **Without Anatomy PNB** | **z** | **p** | **n** |
| --- | --- | --- | --- | --- | --- |
| **Adductor Canal** | 82.64 | 79.80 | 0.03521 | 0.9681 | 21 |
| **Axillary** | 82.27 | 60.50 | 0.45772 | 0.6455 | 21 |
| **ESP** | 50.40 | 32.73 | -1.40836 | 0.15854 | 21 |
| **Interscalene** | 106.00 | 134.40 | -1.13389 | 0.25848 | 20 |
| **Popliteal** | 106.30 | 105.91 | -0.52813 | 0.59612 | 21 |
| **Rectus Sheath** | 31.09 | 34.90 | 0.10563 | 0.9124 | 21 |
| **Average** | **75.92** | **74.53** |  |  |  |

No statistically significant difference (Mann-Whitney U test) was observed in average scan time for any of the blocks.

## Participant confidence – by block

### Interscalene

| **Participant Confidence** | **With Anatomy PNB** | **Without Anatomy PNB** | **TOTAL SCANS** |
| --- | --- | --- | --- |
| **0** | 0 | 2 | 2 |
| **1** | 1 | 0 | 1 |
| **2** | 1 | 2 | 3 |
| **3** | 0 | 1 | 1 |
| **4** | 0 | 1 | 1 |
| **5** | 0 | 1 | 1 |
| **6** | 1 | 1 | 2 |
| **7** | 2 | 1 | 3 |
| **8** | 4 | 2 | 6 |
| **9** | 1 | 0 | 1 |
| **TOTAL SCANS** | 10 | 11 | 21 |

A Mann-Whitney U test showed there was a statistically significant increase in participant confidence scores when scanning the interscalene block with Vs without Anatomy PNB (W = 20.5, N = 21, p = 0.0128).

### Axillary

| **Participant Confidence** | **WITH ANATOMY PNB** | **WITHOUT ANATOMY PNB** | **TOTAL SCANS** |
| --- | --- | --- | --- |
| **3** | 0 | 1 | 1 |
| **4** | 1 | 0 | 1 |
| **5** | 1 | 1 | 2 |
| **6** | 4 | 0 | 4 |
| **7** | 2 | 5 | 7 |
| **8** | 3 | 3 | 6 |
| **TOTAL SCANS** | 11 | 10 | 21 |

A Mann-Whitney U test showed there was no statistically significant difference between the participant confidence scores when scanning the axillary block with or without Anatomy PNB (W = 41, N = 21, p = 0.3232).

### ESP

| **Participant Confidence** | **With Anatomy PNB** | **Without Anatomy PNB** | **TOTAL SCANS** |
| --- | --- | --- | --- |
| **6** | 3 | 1 | 4 |
| **7** | 1 | 3 | 4 |
| **8** | 2 | 5 | 7 |
| **9** | 3 | 2 | 5 |
| **10** | 1 | 0 | 1 |
| **TOTAL SCANS** | 10 | 11 | 21 |

A Mann-Whitney U test showed there was no statistically significant difference between the participant confidence scores when scanning the ESP block with or without Anatomy PNB (W = 70, N = 21, p = 0.2842).

### Rectus Sheath

| **Participant Confidence** | **With Anatomy PNB** | **Without Anatomy PNB** | **TOTAL SCANS** |
| --- | --- | --- | --- |
| **5** | 0 | 1 | 1 |
| **7** | 1 | 4 | 5 |
| **8** | 7 | 3 | 10 |
| **9** | 3 | 1 | 4 |
| **10** | 0 | 1 | 1 |
| **TOTAL SCANS** | 11 | 10 | 21 |

Table 32: Participant self-rated confidence in their achieved block site - Rectus Sheath

A Mann-Whitney U test showed there was no statistically significant difference between the participant confidence scores when scanning the rectus sheath block with or without Anatomy PNB, W = 46.5, N = 21, p = 0.5390.

### Adductor Canal

| **Participant Confidence** | **With Anatomy PNB** | **Without Anatomy PNB** | **TOTAL SCANS** |
| --- | --- | --- | --- |
| **0** | 1 | 0 | 1 |
| **2** | 0 | 1 | 1 |
| **4** | 0 | 2 | 2 |
| **5** | 1 | 0 | 1 |
| **6** | 1 | 1 | 2 |
| **7** | 2 | 0 | 2 |
| **8** | 4 | 5 | 9 |
| **9** | 2 | 0 | 2 |
| **10** | 0 | 1 | 1 |
| **TOTAL SCANS** | 11 | 10 | 21 |

A Mann-Whitney U test showed there was no statistically significant difference between the participant confidence scores when scanning the adductor canal block with or without Anatomy PNB (W = 50, N = 21, p = 0.7482).

### Popliteal

| **Participant Confidence Score** | **With Anatomy PNB** | **Without Anatomy PNB** | **TOTAL SCANS** |
| --- | --- | --- | --- |
| **3** | 0 | 1 | 1 |
| **4** | 2 | 1 | 3 |
| **6** | 3 | 4 | 7 |
| **7** | 3 | 3 | 6 |
| **8** | 2 | 2 | 4 |
| **TOTAL SCANS** | 10 | 11 | 21 |

A Mann-Whitney U test showed there was no statistically significant difference between the participant confidence scores when scanning the popliteal block with or without Anatomy PNB (W = 49, N = 21, p = 0.6926).

## Expert observer’s assessment of the participant’s total scanning performance (global scan score) – by block

### Interscalene

| **Global Score** | **With Anatomy PNB** | **Without Anatomy PNB** | **TOTAL SCANS** |
| --- | --- | --- | --- |
| **0** | 0 | 5 | 5 |
| **3** | 1 | 0 | 1 |
| **4** | 0 | 1 | 1 |
| **5** | 1 | 0 | 1 |
| **6** | 1 | 3 | 4 |
| **7** | 6 | 2 | 8 |
| **8** | 1 | 0 | 1 |
| **TOTAL SCANS** | 10 | 11 | 21 |

A Mann-Whitney U test showed there was a statistically significant increase in global scan scores when scanning the interscalene block with Vs without Anatomy PNB , W = 20.5, N = 21, p = 0.0128.

### Axillary

| **Global Score** | **With Anatomy PNB** | **Without Anatomy PNB** | **TOTAL SCANS** |
| --- | --- | --- | --- |
| **2** | 0 | 1 | 1 |
| **3** | 0 | 1 | 1 |
| **4** | 0 | 1 | 1 |
| **5** | 1 | 0 | 1 |
| **6** | 5 | 3 | 8 |
| **7** | 2 | 3 | 5 |
| **8** | 3 | 1 | 4 |
| **TOTAL SCANS** | 11 | 10 | 21 |

A Mann-Whitney U test showed there was no statistically significant difference between the global scan scores when scanning the axillary block with or without Anatomy PNB (W = 41, N = 21, p = 0.3232).

### ESP

| **Global Score** | **With Anatomy PNB** | **Without Anatomy PNB** | **TOTAL SCANS** |
| --- | --- | --- | --- |
| **6** | 2 | 0 | 2 |
| **7** | 3 | 3 | 6 |
| **8** | 3 | 5 | 8 |
| **9** | 2 | 3 | 5 |
| **TOTAL SCANS** | 10 | 11 | 21 |

A Mann-Whitney U test showed there was no statistically significant difference between the global scan scores when scanning the ESP block with or without Anatomy PNB (W = 70, N = 21, p = 0.2842).

### Rectus Sheath

| **Global Score** | **With Anatomy PNB** | **Without Anatomy PNB** | **TOTAL SCANS** |
| --- | --- | --- | --- |
| **6** | 1 | 1 | 2 |
| **7** | 2 | 3 | 5 |
| **8** | 6 | 5 | 11 |
| **9** | 2 | 1 | 3 |
| **TOTAL SCANS** | 11 | 10 | 21 |

A Mann-Whitney U test showed there was no statistically significant difference between the global scan scores when scanning the rectus sheath block with or without Anatomy PNB (W = 46.5, N = 21, p = 0.5390).

### Adductor Canal

| **Global Score** | **With Anatomy PNB** | **Without Anatomy PNB** | **TOTAL SCANS** |
| --- | --- | --- | --- |
| **2** | 1 | 0 | 1 |
| **3** | 1 | 2 | 3 |
| **4** | 0 | 2 | 2 |
| **5** | 1 | 1 | 2 |
| **6** | 2 | 1 | 3 |
| **7** | 3 | 1 | 4 |
| **8** | 3 | 2 | 5 |
| **9** | 0 | 1 | 1 |
| **TOTAL SCANS** | 11 | 10 | 21 |

A Mann-Whitney U test showed there was no statistically significant difference between the global scan scores when scanning the adductor canal block with or without Anatomy PNB (W = 50, N = 21, p = 0.7482).

### Popliteal

| **Global Score** | **With Anatomy PNB** | **Without Anatomy PNB** | **TOTAL SCANS** |
| --- | --- | --- | --- |
| **2** | 0 | 1 | 1 |
| **3** | 1 | 1 | 2 |
| **4** | 0 | 2 | 2 |
| **5** | 2 | 1 | 3 |
| **6** | 4 | 2 | 6 |
| **7** | 1 | 1 | 2 |
| **8** | 2 | 3 | 5 |
| **TOTAL SCANS** | 10 | 11 | 21 |

A Mann-Whitney U test showed there was no statistically significant difference between the global scan scores when scanning the popliteal block with or without Anatomy PNB, W = 49, N = 21, p = 0.6926.
